# A method to design a fast chaotic oscillator using CCTA

**DOI:** 10.1016/j.mex.2024.102801

**Published:** 2024-06-12

**Authors:** Chandan Kumar Choubey, Aruna Pathak, Manoj Kumar Tiwari

**Affiliations:** a Symbiosis Institute of Technology, Pune Campus, Symbiosis International (Deemed University), Pune, India; b Department of Electronics and Telecommunication Engineering, Government Engineering College Bharatpur, Rajasthan, India; c Department of Physical IP and Mixed Signal Design Solutions (TR&D), STMicroelectronics Pvt. Ltd., Greater Noida, India

**Keywords:** Analog circuit, Chua's circuit, Fast oscillation, Non-linear resistor, Negative resistance, Analog building block, Design a fast chaotic Oscillator using CCTA

## Abstract

This article introduces a novel method for designing a fast chaotic oscillator using a CCTA (Current Conveyor Transconductance Amplifier) based on Chua's circuit. The proposed method uses innovative configurations and advanced simulation techniques to overcome challenges in high-speed operation, nonlinear dynamics, and Analog Building Block (ABB) selection. The design begins with nonlinear negative resistance, essential for Chua's diode characteristics, including two negative resistances, NR_1_ and NR_2_. The circuit integrates one CCTA block, two grounded capacitors, two fixed resistors, one inductor, and one potentiometer. It is simulated using PSPICE with IC (Integrated Circuit) macro-models and 180nm CMOS (Complementary Metal Oxide Semiconductor) technology. Various chaotic waveforms and attractors are produced, validating the theoretical and mathematical predictions. By varying the resistance values (1450Ω, 1650Ω, 1800Ω, 1950Ω), the circuit exhibits different chaotic behaviors, such as large limit cycles, double-scroll attractors, Rossler-type attractors, and I-periodic attractors. FFT (Fast Fourier Transform) analysis confirms the highest dominant operating frequency of 37.5MHz. A Monte Carlo simulation with 100 runs shows maximum voltage variations in the chaotic waveforms of 5.21 % and 4.61 % across the capacitors, demonstrating robustness and reliability. This design offers significant advancements in implementing high-frequency chaotic oscillators, with potential applications in various fields requiring chaotic signal generation.

• A novel design of Chua's diode and Chua's chaotic oscillator using only one CCTA block is presented in this paper.
• The proposed chaotic oscillator achieves the highest operating frequency of 37.5MHz.
• The proposed circuit is simulated using commercially available ICs (MAX435 and AD844) and CMOS 180nm technology in PSPICE to confirm its workability.

## Graphical abstract

**Figure:**
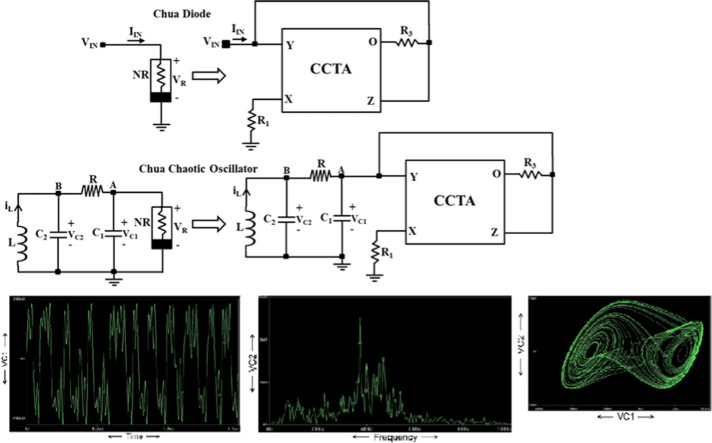

Specifications table

**Table:** 

Subject area:	Engineering
More specific subject area:	Electronics circuit design
Name of your method:	Design a fast-chaotic Oscillator using CCTA
Name and reference of original method:	M. P. Kennedy, “Robust op-amp realization of Chua's circuit,” Frequenz, vol. 46, no. (3–4), pp. 66–80, 1992. https://doi.org/10.1515/FREQ.1992.46.3–4.66
Resource availability:	N.A

## Background

**Fig. 1:**
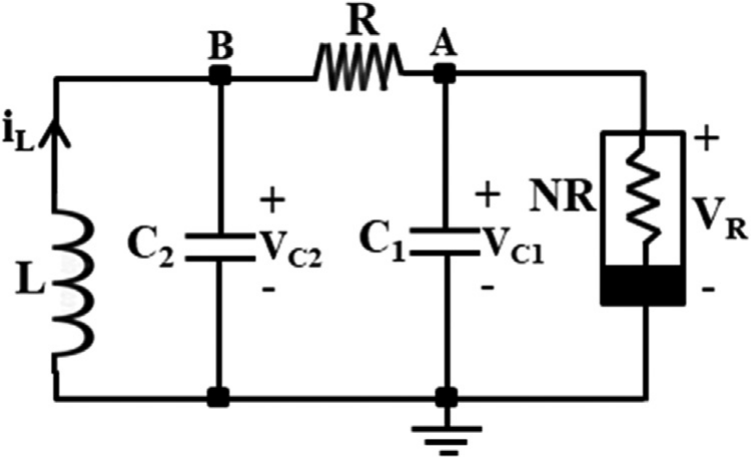
Circuit diagram for the traditional Chua's circuit [7].

**Fig. 2:**
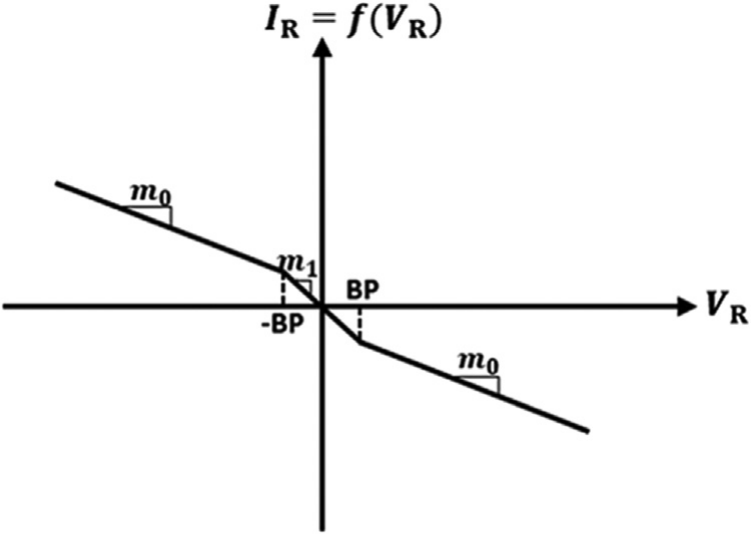
V-I Characteristic of NR [7].

Chaotic signals are widely acknowledged for their multiple benefits, encompassing secure communication [1], heart rate assessment [2], circuit stabilization [3], packet switching in computer networks [4], chaos management in robotic devices [5], and visual sensing [6]. Chua's circuit is a renowned and simple chaotic oscillator that can produce chaos, and it has attracted the attention of researchers in the domain of nonlinear dynamical circuits and systems. Fig. 1 shows the schematic of Chua's circuit [7], which comprises one linear resistor (R), one linear inductor (L), two linear capacitors (C_1_ and C_2_), and one nonlinear resistor (NR), also called Chua's diode [7]. The terms (V_C1_ and V_C2_) are voltages across capacitors (C_1_ and C_2_), respectively, and i_L_ is the current flow through the inductor (L). Chua's diode is the essential component of Chua's circuit since it imparts nonlinear qualities to the system. Generally, a Chua's diode has been implemented by connecting two voltage-controlled negative impedance converters in parallel [7]. This type of implementation employs at least two typical voltage-mode op-amps and many resistors. The V-I characteristics of Chua's diode is depicted in Fig. 2, which shows the non-linearity with negative slopes. In Fig. 2, m_1_ and m_0_ correspond to the slopes of the inner and outer straight lines, respectively, while ±BP denotes the breakpoints [7]. Due to the voltage-mode blocks like op-amp and more resistors, Chua's circuit's operating frequency is restricted to a few kHz ranges. Therefore, an improved version of the chaotic oscillator using a high-performance current-mode block and few resistors is proposed, which runs faster and achieves the highest operating frequency of 37.5MHz.

Numerous research articles on chaotic electronic circuit realizations of well-known dynamical systems in mathematics have been reported. These circuits can be called voltage-mode chaotic circuits because many use voltage-mode blocks like op-amp as active elements. However, voltage-mode circuits suffer from low slew rates and fixed gain-band products, which substantially impact the operating frequency of voltage-mode chaotic circuits [8]. At the same time, literature witnesses the transferral of analog integrated circuit implementation from voltage-mode to current-mode circuits, whereby transmissible signals are currents rather than voltages. The current-mode equivalents have low power consumption, a high slew rate, and wide signal bandwidth compared to their voltage-mode counterparts [9,10].

Designing a chaotic oscillator presents numerous significant difficulties and challenges that must be addressed with precision and ingenuity. One such challenge involves achieving high-speed operation, necessitating careful design considerations to ensure signal integrity and stability at such fast speeds. Another critical aspect is the selection of an Analog Building Block (ABB), where choosing components with the required bandwidth, linearity, and noise performance is crucial. Furthermore, accurately simulating the circuit behavior, including nonlinear dynamics and high-frequency effects, is challenging. Addressing these challenges requires a multidisciplinary approach involving expertise in analog design, nonlinear dynamics, signal processing, and experimental validation.

Several studies have been conducted to develop different Chua's circuit architectures as a chaotic model. Most of the methods revolve around the design of a simple Chua's diode, also called Non-linear Resistor (NR), because it is the essential component of the Chua circuit, responsible for non-linearity. As a result, different Chua's oscillator circuits have centered on the straightforward and efficient Chua's diode, employing fewer active and passive electrical components.

The Chua's diode, or NR, a vital component of a Chua's circuit, has undergone extensive modifications for smoother linearity and cubic linearity [11]. Chua's diode using various ABBs is well documented in the literature by using Current Conveyor [12], Current-Controlled Current Conveyor Feedback Amplifier (CCCFA) [13], Current Differential Transconductance Amplifier (CDTA) [14], Extra-X Current Controlled Current Conveyor (EXCCCII) [15], Current Controlled Current Conveyor Transconductance Amplifier (CCCCTA) [16], Voltage Differencing Transconductance Amplifier (VDTA) [17], Voltage-Differencing Gain Amplifier (VDGA) [18], and many others. Other feasible alternatives may be the use of a voltage-mode operational amplifier in the Chua circuit, which limits the performance in terms of frequency and linearity [[19], [20], [21]].

To overcome the major difficulties and challenges associated with design, the proposed research likely introduced several original achievements. The research likely introduced a novel circuit topology tailored to meet the requirements of high-speed operation and nonlinear dynamics. This topology incorporates innovative configurations of active and passive components to achieve the desired performance characteristics. Advanced simulation techniques have been employed to accurately model the oscillator's behavior, including nonlinear dynamics. One of the primary achievements would be achieving the targeted high operating frequency while maintaining stable and reliable chaotic behavior.

The evidence mentioned above sheds light on the fantastic phenomena of Chua's circuit and enables the implementation of Chua's circuit using various active building blocks. One of the most adaptable and practical current-mode building blocks is the Current Conveyor Transconductance Amplifier (CCTA) [[22], [23], [24], [25], [26]].

The CCTA has a number of important benefits, including increased dynamic range, wide bandwidth, high linearity, high input impedance, low output impedance, adaptability in design, temperature stability, ease of integration, and versatility that is useful in filtering, oscillation, and signal transformation tasks [[22], [23], [24], [25], [26]].

Adopting a CCTA-based approach to build the Fast Chaotic Oscillator marks a significant advancement in circuit design. Key highlights of this innovation include the introduction of a novel Chua's diode and Chua's chaotic oscillator, achieved with just one CCTA block, showcasing efficiency and simplicity. CCTAs have strong linearity, enhancing accuracy and fidelity in chaotic signal production. Compared to alternative approaches, this can result in more steady and predictable chaotic behavior. Notably, the CCTAs are also renowned for their fast operation and large bandwidth, which allow the oscillator to reach the desired operating frequency of 37.5MHz. This makes it possible to interpret signals more quickly and improves performance in high-frequency applications.

Due to this device's increasing interest, many analog function circuits have been developed. This research article proposes a single CCTA-based Chua's diode that can generate well-known chaotic attractors. It gives a chance to investigate the ongoing developments in current-mode circuits.

The research innovation of the proposed Fast Chaotic Oscillator represents the novelty of the circuit by overcoming the research gaps in the literature. The circuit's novelty is underlined by several key features. Firstly, a novel design of Chua's diode and Chua's chaotic oscillator using only one CCTA block is presented in this paper. The proposed chaotic oscillator achieves the highest operating frequency of 37.5 MHz. Simulation results further validate the circuit's capabilities, with the development of chaotic attractors such as large limit cycle, double-scroll attractor, Rossler-type attractor, and I-periodic attractor, affirming theoretical and mathematical predictions. Practical feasibility is confirmed through simulations using commercially available ICs (MAX435 and AD844) and CMOS 180 nm technology in PSPICE. Additionally, a Monte Carlo simulation is conducted to assess the uncertainty and robustness of the proposed Chua chaotic circuit, further establishing its reliability and applicability in real-world scenarios.

## Method details

The equations involved in the traditional chaotic oscillator, shown in Fig. 1, are discussed first in this section, followed by the method to design the proposed CCTA-based fast chaotic oscillator.

The following differential equations govern the dynamics of the Chua's circuit of Fig. 1 [7].

(1) $$RC_{1}\frac{dV_{C1}}{dt}=\left(V_{C2}-V_{C1}\right)-Rg\left(V_{R}\right)$$

(2) $$RC_{2}\frac{dV_{C2}}{dt}=\left(V_{C1}-V_{C2}\right)+R\left(I_{L}\right)$$

(3) $$L\frac{dI_{L}}{dt}=-V_{C2}$$

(4) $$gV_{R}=m_{0}V_{R}+0.5\left(m_{1}-m_{0}\right)\times \;\left[\left|V_{R}+\;V_{BP}\right|-\left|V_{R}-\;V_{BP}\right|\right]$$

where voltage through C_1_, voltage through C_2_, and current via L correspond to state variables V_C1_, V_C2_, and $I_{L}$ respectively. The slopes of the outer and inner areas are represented by m_0_ and m_1_, respectively. ±V_BP_ signifies the breakpoints presented in Fig. 2 for a piecewise linear NR in Chua's circuit.

The Chua's circuit is a third-order chaotic oscillator whose characteristic equation is defined as [17]:

(5) $$s^{3}+s^{2}\;\left[\frac{G}{C_{2}}+\frac{G+m_{i}}{C_{1}}\;\right]+\;s\;\left[\frac{1}{LC_{2}}+\frac{Gm_{i}}{C_{1}C_{2}}\;\right]+\left[\frac{G+m_{i}}{LC_{1}C_{2}}\;\right]=0$$

From Eq. (5), the CO (Condition of Oscillation) and FO (Frequency of Oscillation) can be obtained as [17]:

(6) $$CO:\;R=-\frac{Lm_{i}\left(C_{1}+C_{2}\right)}{C_{1}^{2}+LC_{2}m_{i}^{2}},\;\left|\frac{Lm_{i}}{RC_{1}}\right|<1$$

(7) $$FO:\;f_{o}=\frac{1}{2\pi \sqrt{LC_{2}}}\;\sqrt{1+\frac{Lm_{i}}{RC_{1}}},\;i=0,\;1$$

### Current conveyor transconductance amplifier CCTA

**Fig. 3:**
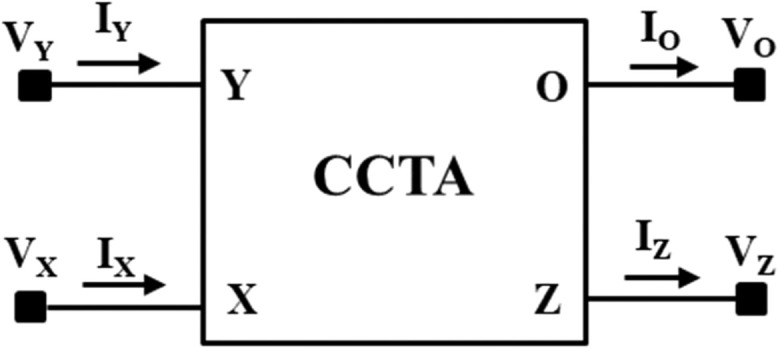
Circuit symbol of CCTA [22].

**Fig. 4:**
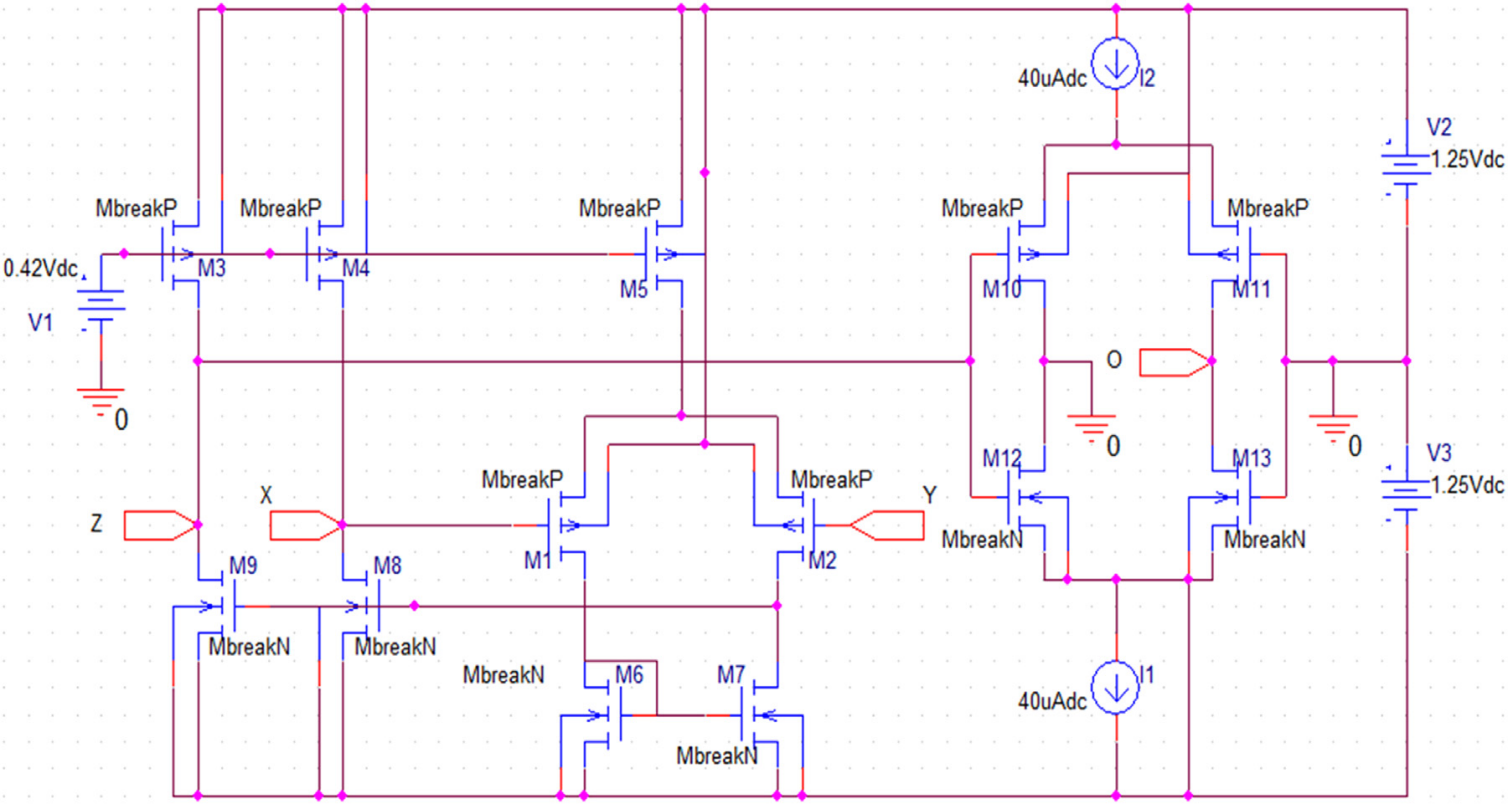
A CMOS implementation of CCTA.

**Fig. 5:**
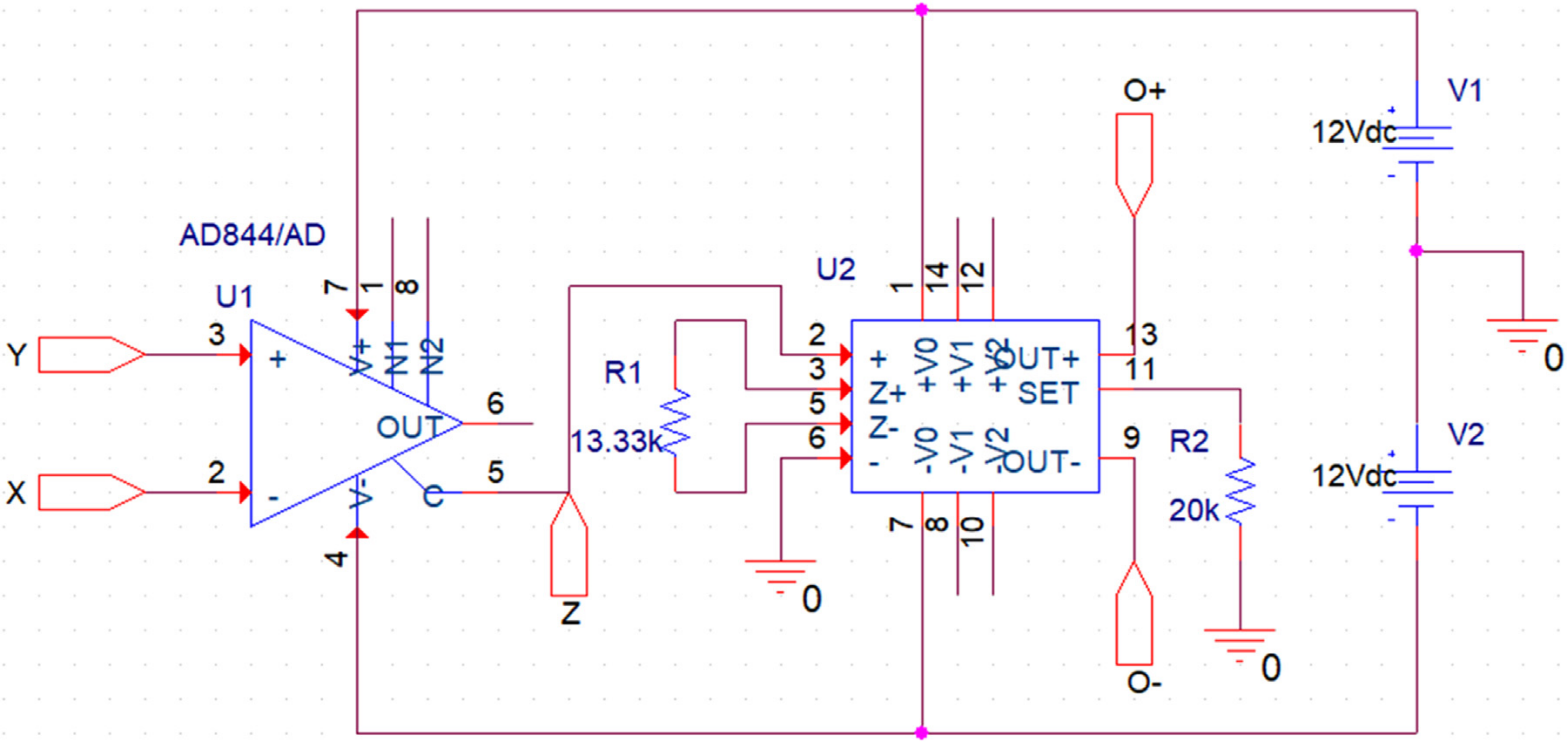
An IC-based implementation of CCTA.

The Current Conveyor Transconductance Amplifier (CCTA) [22] is a versatile current-mode block that can work both in Voltage-Mode (VM) and Current-Mode (CM) circuits. The circuit symbol of the CCTA is shown in Fig. 3. It has four Input/output (I/O) terminals. A possible CMOS implementation and an IC-based implementation of it, which are used in the simulation, are depicted in Fig. 4, Fig. 5, respectively. In Fig. 4, a CMOS implementation of the Second-Generation Current Conveyor (CCII) is cascaded with the Arbel-Goldminz CMOS cell of the Operational Transconductance Amplifier (OTA) for implementing the CMOS-based CCTA. The gate terminals of Metal Oxide Semiconductor Field Effect Transistors (MOSFETs), M3, M4, and M5, are fed by the voltage source V1. The current sources, I1 and I2, determine the transconductance gain g_m_. In Fig. 5, two high-performance ICs, AD844 and MAX435, are cascaded to implement the IC-based CCTA. The AD844 is a Current Feedback Operational Amplifier (CFOA) IC manufactured by Analog Devices INC., whereas the MAX435 is an OTA IC manufactured by Maxim Integrated. The resistor R_1_ of Fig. 5 limits the bias current and hence sets the g_m_, whereas the resistor R_2_ sets the supply current of the IC MAX435.

The electrical characteristics of CCTA are as follows [22]:

(8) $$I_{Y}=0;\;V_{X}=V_{Y};\;I_{Z}=I_{X};\;I_{O}=g_{m}V_{Z}$$

### Implementation of Chua diode

A CCTA-based straightforward Chua diode is implemented using two negative resistances, NR_1_ and NR_2_. Negative resistance is a special type of synthetic resistance that exhibits a negative slope in its V-I characteristics. The implementation of NR_1_, NR_2_, and Chua diode is discussed as follows.

### Implementation of NR_1_

**Fig. 6:**
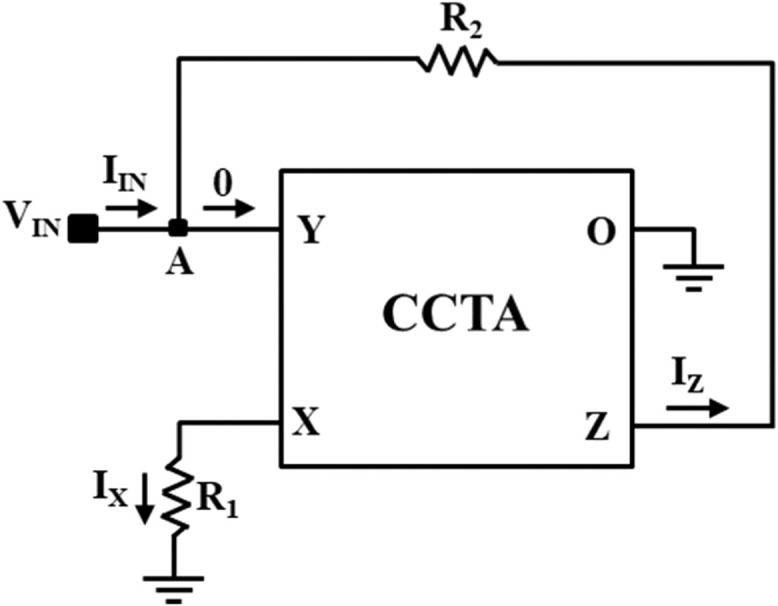
NR_1_ implementation using CCTA.

**Fig. 7:**
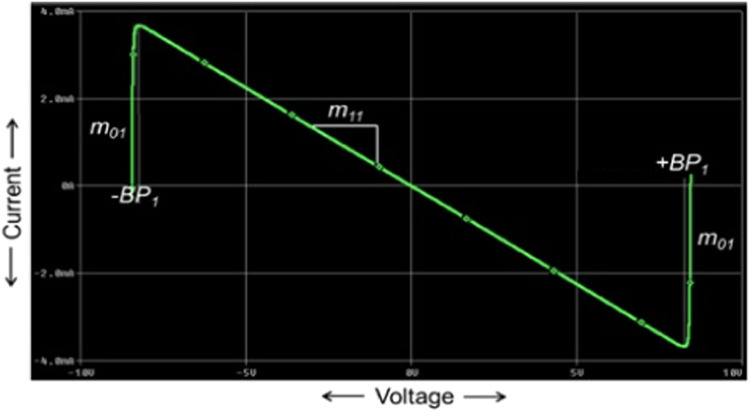
V-I characteristics of NR_1_.

NR_1_ is implemented using one CCTA block and two resistors, R_1_ and R_2_, as illustrated in Fig. 6. The V-I characteristics of NR_1_ is obtained from the DC sweep analysis of NR_1_ using IC-based CCTA. This is depicted in Fig. 7. The V-I characteristic curve has three distinct regions separated by two symmetrically placed breakpoints (±BP_1_). The central region between the two breakpoints has a negative slope (m_11_), while the other two outer regions have positive slopes (m_01_). The slopes in these regions represent the rates of change of current with respect to voltage. A three-segment non-linear charatersitics with negative inner slope m_11_, outer slope m_01_, and breakpoints ±V_BP1_ can be observed in Fig. 7.

Inner slope m_11_, outer slope m_01_, and breakpoints ±BP_1_ are discussed and formulized as follows:

### Calculation of inner slope (m_11_)

On applying the port-relationship of the CCTA in the Fig. 6, current I_X_ can be expressed as:

(9) $$I_{X}=\frac{V_{X}}{R_{1}}=\frac{V_{IN}}{R_{1}}$$

Again, by using the port relationship of CCTA, current I_Z_ can be written as:

(10) $$I_{Z}=\frac{V_{IN}}{R_{1}}$$

On applying KCL (Kirchhoff's Current Law) at node A in Fig. 6, we get

(11) $$I_{IN}=-I_{Z}$$

On combinig (10) and (11), the internal slope m_11_ can be expressed as:

(12) $$m_{11}=\frac{I_{IN}}{V_{IN}}=-\frac{1}{R_{1}}$$

### Calculation of external slope m_01_ in the saturation region

On applying KVL (Kirchhoff's Voltage Law) in Fig. 6, the input current I_IN_ can be rewritten as:

(13) $$I_{IN}=\frac{V_{IN}-V_{Z}}{R_{2}}=\frac{V_{IN}}{R_{2}}-\frac{V_{Z}}{R_{2}}$$

In saturation region, the voltage at Z-port reaches to the saturation values as $V_{Z}=\pm V_{sat}$. Therefore, (13) can be rewritten as:

(14) $$I_{IN}=\frac{V_{IN}}{R_{2}}-\frac{\pm V_{sat}}{R_{2}}$$

Eq. (14) is an equation of straight line having a positive slope that can be expressed as:

(15) $$m_{01}=\frac{1}{R_{2}}$$

### Calculation of the first breakpoint voltages (±V_BP1_)

Again, applying KVL in Fig. 6, V_Z_ can be expressed as:

(16) $$V_{Z}=V_{IN}+I_{Z}R_{2}$$

By putting the value of I_Z_ From Eq. (10) in Eq. (16), we get

(17) $$V_{IN}=\frac{V_{Z}}{1+\frac{R_{2}}{R_{1}}}$$

At the boundary of the saturation region, $V_{Z}=\pm V_{sat}$ and $V_{IN}=\pm V_{BP1}$, then (17) can be rewritten as:

(18) $$\pm V_{BP1}=\frac{\pm V_{sat}}{1+\frac{R_{2}}{R_{1}}}$$

### Implementation of NR_2_

**Fig. 8:**
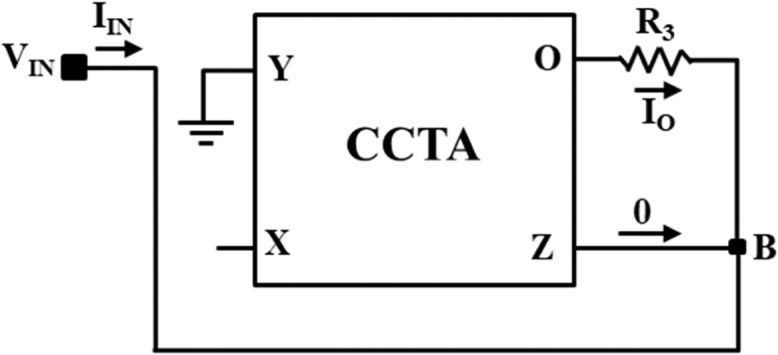
NR_2_ implementation using CCTA.

**Fig. 9:**
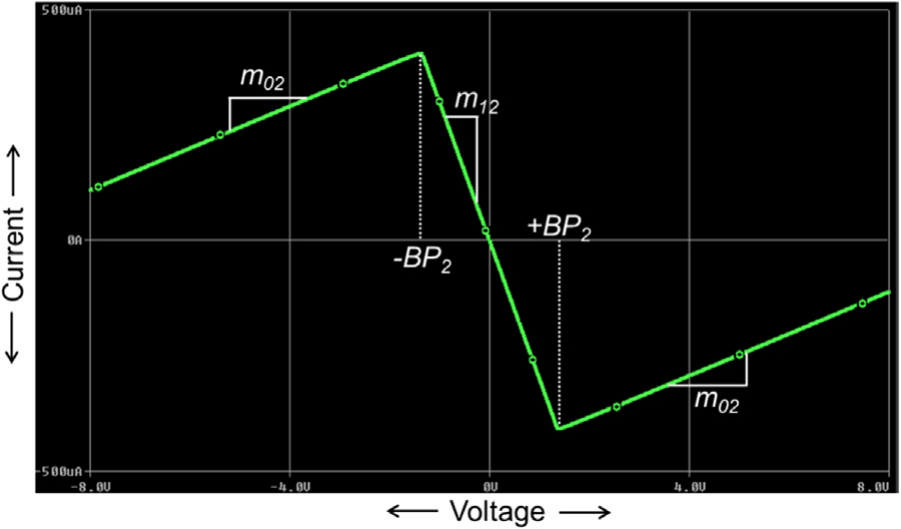
V-I characteristics of NR_1_.

Now, the NR_2_ is implemented using one CCTA block and one resistor, R_3_, as illustrated in Fig. 8. Similar to NR_1_, the V-I characteristics of NR_2_ are obtained from the DC sweep analysis using IC-based CCTA. This is depicted in Fig. 9, having three distinct regions separated by two symmetrically placed breakpoints (±BP_2_). The central region between the two breakpoints has a negative slope (m_12_), while the other two outer regions have positive slopes (m_02_). The slopes in these regions represent the rates of change of current with respect to voltage.

A three-segment non-linear characteristic with negative inner slope m_12_, outer slope m_02_, and breakpoints ±BP_2_ can be observed in Fig. 9.

### Calculation of internal slope m_12_

On applying KCL at node B in Fig. 6, we get

(19) $$I_{IN}=-I_{O}$$

using the port-relationship of CCTA in Eq. (12), we get

(20) $$I_{IN}=-g_{m}V_{IN}$$

From Eq. (20), the innerl slope m_12_ can be expressed as:

(21) $$m_{12}=\frac{I_{IN}}{V_{IN}}=-g_{m}$$

### Calculation of external slope m_02_

On applying KVL in Fig. 6, the input current I_IN_ can be expressed as:

(22) $$I_{IN}=\frac{V_{IN}-V_{O}}{R_{3}}=\frac{V_{IN}}{R_{3}}-\frac{V_{O}}{R_{2}}$$

In the saturation region, the voltage at O-port reaches to the saturation values as $V_{O}=\pm V_{sat}$. Therefore Eq. (22) can be rewritten as:

(23) $$I_{IN}=\frac{V_{IN}}{R_{3}}-\frac{\pm V_{sat}}{R_{3}}$$

Here, it can be noticed that Eq. (16) is an equation of a straight line having a slope m_02_:

(24) $$m_{02}=\frac{1}{R_{3}}$$

### Calculation of the second breakpoint voltages (±V_BP2_)

Again, on applying KVL in Fig. 6, the voltage of port-O can be expressed as:

(25) $$V_{O}=V_{IN}+I_{O}R_{3}$$

Using the port relationship of Eq. (3) in Eq. (25), we get

(26) $$V_{IN}=\frac{V_{O}}{1+g_{m}R_{3}}$$

At the boundary of the saturation region, $V_{O}=\pm V_{sat}$ and $V_{IN}=\pm V_{BP2}$, then (26) can be rewritten as:

(27) $$\pm V_{BP2}=\frac{\pm V_{sat}}{1+g_{m}R_{3}}$$

### Implementation of Chua diode

**Fig. 10:**
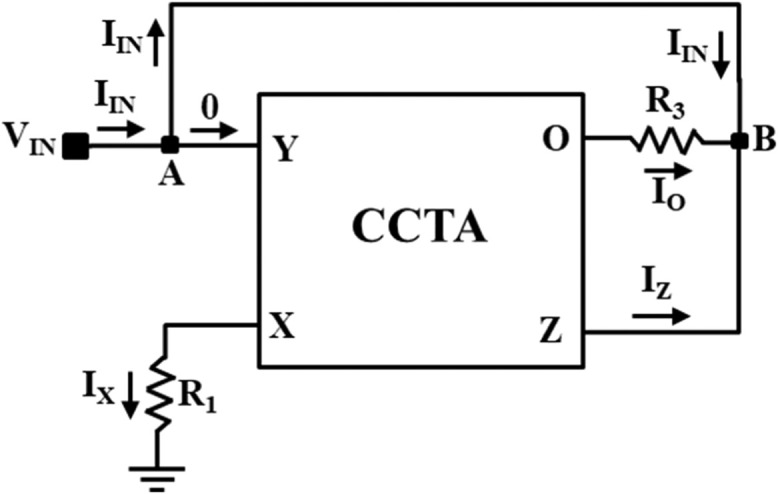
Chua diode implementation using a single CCTA.

The proposed CCTA-based Chua diode has been designed by careful combining of the NR_1_ and NR_2_, as shown in Fig. 10. It has a negative slope (m_1_) and positive slopes (m_0_). The slopes represent the rates of change of current with respect to voltage. While combining the NR_1_ and NR_2_, the resistor R_2_ is intentionally removed (replaced by a short circuit) from the design as it always operates in the saturation region. Also, only one CCTA block has been used instead of two. As R_2_ = 0, from (15), (18), the outer slope and the breakpoints of NR_1_ can be written as $m_{01}=\infty$ and $\pm V_{BP1}=\pm V_{sat}$, respectively.

By applying the port-relationship of the CCTA in Fig. 10, we get the current at Z-port, I_Z_ as:

(28) $$I_{Z}=I_{X}=\frac{V_{X}}{R_{1}}=\frac{V_{IN}}{R_{1}}$$

Again, using the port-relationship, the current I_O_ can be written as:

(29) $$I_{O}=g_{m}V_{Z}=g_{m}V_{IN}$$

On applying KCL at node B in Fig. 10, we get

(30) $$I_{IN}=-\left(I_{Z}+I_{O}\right)=-\left(\frac{V_{IN}}{R_{1}}+g_{m}V_{IN}\right)$$

From (23), the inner slope (m_1_) of the Chua's diode can be written as:

(31) $$m_{1}=\frac{V_{IN}}{I_{IN}}=-\frac{1}{R_{1}}-g_{m}$$

From (5), (14), and (24), m1 can be expressed as:

(32) $$m_{1}=m_{11}+m_{12}$$

Applying KCL at node B, again I_IN_ can be written as:

(33) $$I_{IN}=-I_{O}-I_{Z}=-\frac{V_{O}-V_{IN}}{R_{3}}-I_{Z}=-\frac{V_{O}}{R_{3}}+\frac{V_{IN}}{R_{3}}-\frac{V_{IN}}{R_{1}}$$

Now, for the saturation region where V_O_ = ±Vsat, IIN can be written as:

(34) $$I_{IN}=\left[-\frac{1}{R_{1}}+\frac{1}{R_{3}}\right]V_{IN}-\frac{\pm V_{sat}}{R_{3}}$$

(27) is a straight line equation, which gives the outer slope (m_0_) as:

(35) $$m_{0}=-\frac{1}{R_{1}}+\frac{1}{R_{3}}$$

From (5), (17), and (28), m1 can be expressed as:

(36) $$m_{0}=m_{11}+m_{02}$$

### Implementation of Chua chaotic oscillator

As the Chua diode introduces the most essential property, non-linearity, into the Chua chaotic oscillator, it becomes a crucial subject for research. Most researchers focus on designing an efficient and straightforward Chua diode using various analog building blocks. Following the same pattern, a CCTA-based Chua diode is designed as shown in Fig. 10. Now, the Chua diode (NR) of the traditional Chua circuit, shown in Fig. 1, is replaced with the proposed CCTA-based Chua diode, as shown in Fig. 12. The final implemented CCTA-based Chua circuit is depicted in Fig. 13. The rest passive elements are used as it is.

## Method validation

**Fig. 11:**
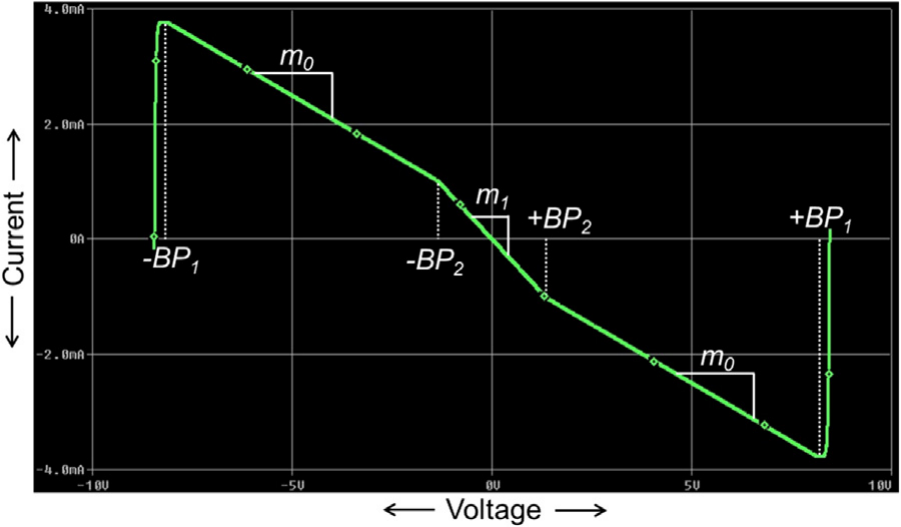
V-I characteristics of the proposed Chua diode.

First, the proposed circuits of NR_1_, NR_2_, and Chua diode are shown in Figs. 6, 8, and 10, respectively, have been simulated in PSPICE using DC sweep analysis to observe the V-I characteristics of these circuits, as shown in Figs. 7, 9, and 11, respectively. In these figures, the respective slopes and breakpoints of the circuits are clearly indicated. An IC-based CCTA implementation, as shown in Fig. 3, has been utilized in this DC sweep analysis. In this implementation, two ICs, AD844 and Max435, are used in a cascaded configuration. As indicated in Fig. 3, the values of resistances and power supplies are taken as R1 = 13.33kΩ, R2 = 20kΩ, V1=+12V, and V2 = −12V. Resistance R1 fixes the value of g_m_ according to the relationship gm = 4/R1, and resistance R2 fixes the supply currents through the IC Max435.

**Fig. 12:**
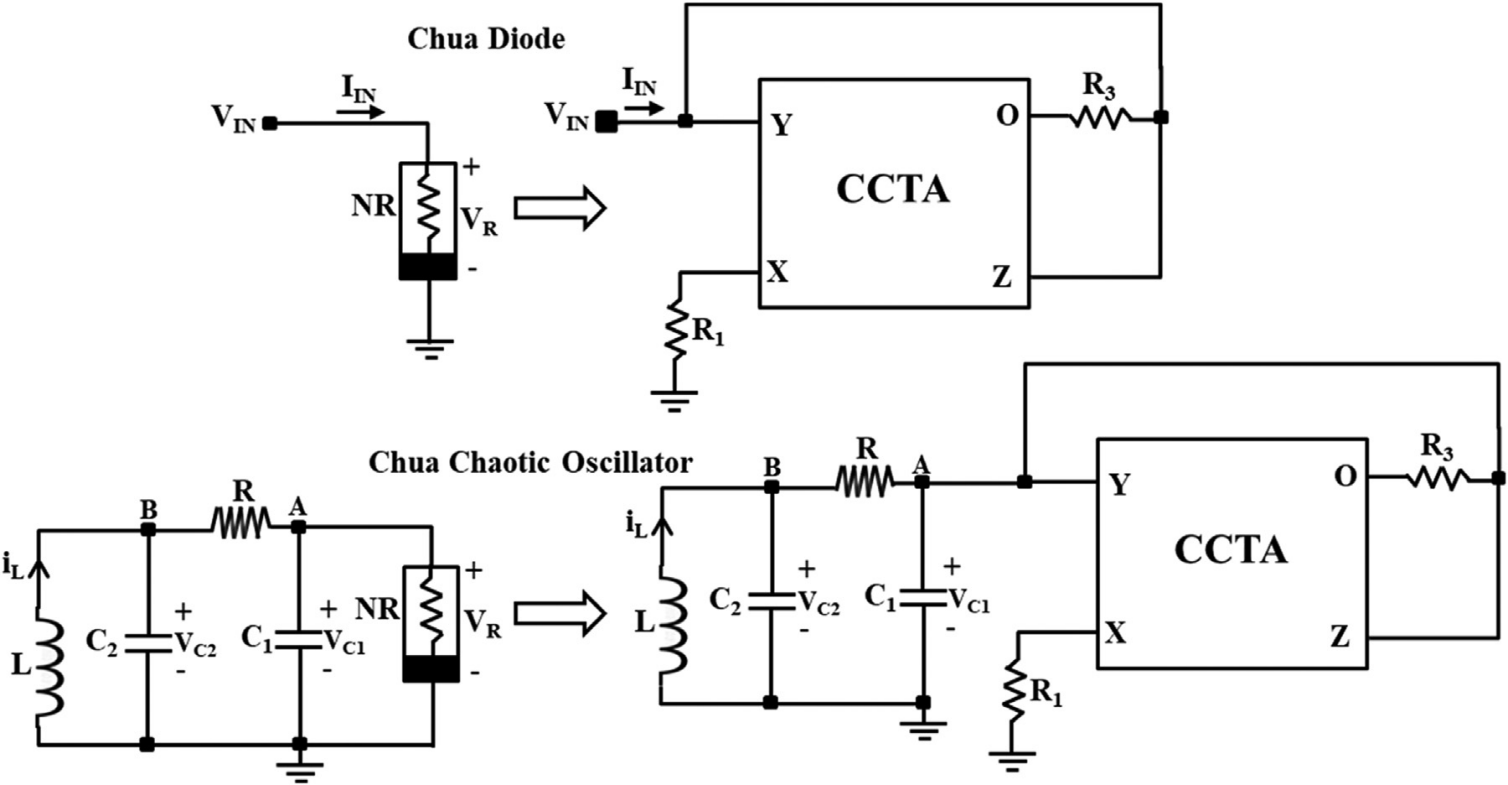
Methodology to design CCTA-based Chua Chaotic Oscillator.

**Fig. 13:**
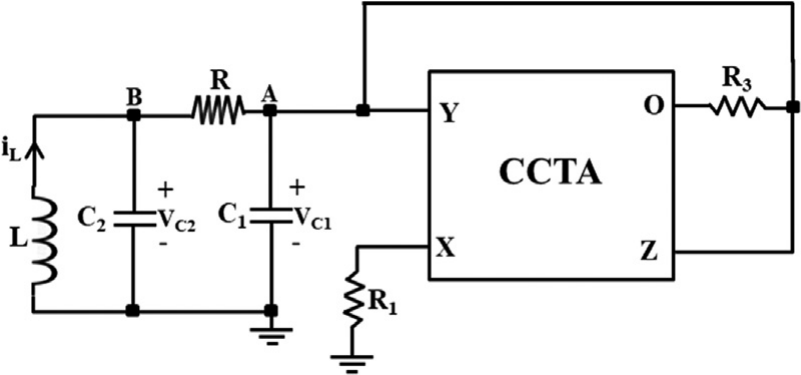
Proposed CCTA-based Chua Chaotic Oscillator.

Further, the proposed CCTA-based Chua chaotic oscillator, shown in Fig. 13, is simulated using transient analysis to get the chaotic waveforms and attractors. In the simulation, first, the analog building block of the Chua circuit, CCTA, is implemented using a CMOS schematic, as shown in Fig. 2. For the proper biasing of MOSFETs, ±1.25V power supplies are utilized. A positive DC supply of 0.42V feeds M3, M4, and M5 gate terminals. The transconductance g_m_ of CCTA is set by the bias currents I1 and I2. In this simulation, these bias currents are taken as I1 = I2 = 40µA. The aspect ratio (W/L ratio) of the N-channel Metal Oxide Semiconductor (NMOS) and P-channel Metal Oxide Semiconductor (PMOS) transistors is given in Table 1. It is simulated using 180nm CMOS technology.

**Table 1:** Aspect ratio of NMOS and PMOS transistors used in Fig. 2.

	PMOS Transistor							NMOS Transistor					
Transistors	M1	M2	M3	M4	M5	M10	M11	M6	M7	M8	M9	M12	M13
Width (W) in µm	0.36	0.36	2.4	2.4	2.4	16.64	16.64	0.67	0.67	2.4	2.4	3.6	3.6
Length (L) in µm	0.18	0.18	0.18	0.18	0.18	0.36	0.36	0.18	0.18	0.18	0.18	0.36	0.36

**Fig. 14:**
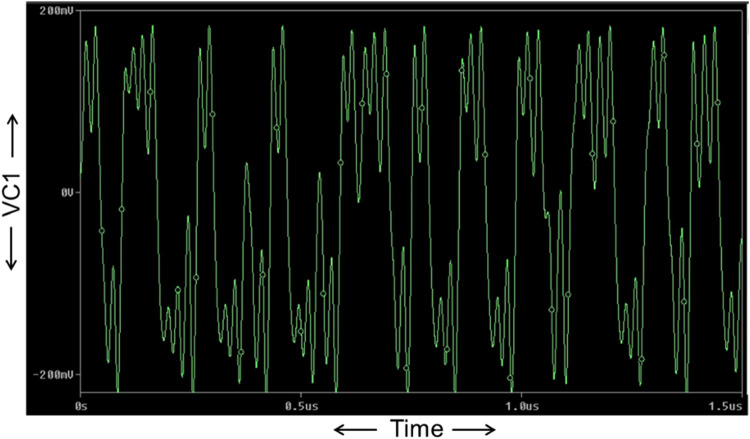
Voltage chaotic waveform generated across capacitor C_1_ of Fig. 13.

**Fig. 15:**
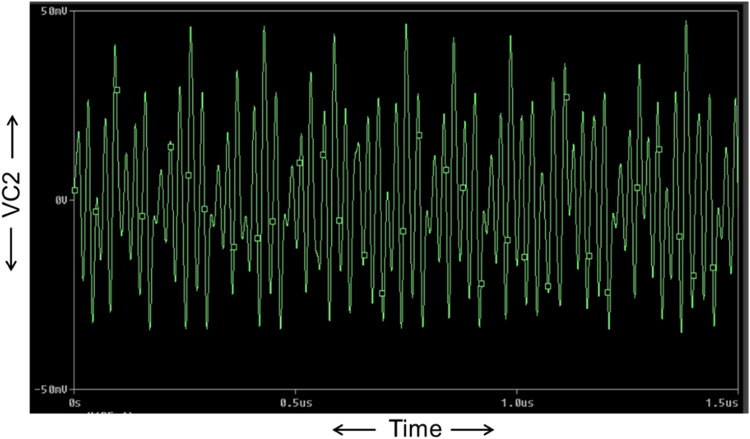
Voltage chaotic waveform generated across capacitor C_2_ of Fig. 13.

**Fig. 16:**
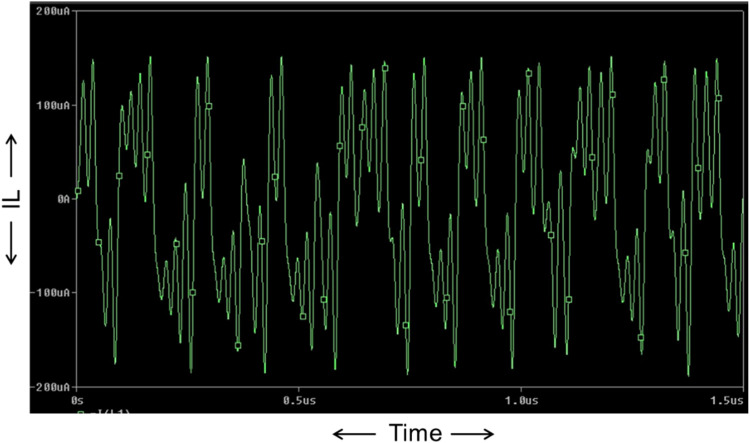
Current chaotic waveform generated through inductor L of Fig. 13.

Now, the proposed Chua chaotic oscillator is designed using CMOS-based CCTA and simulated using 180 nm CMOS technology. The passive components used in the proposed circuit of Fig. 13 are chosen as follows: C_1_ = 0.5pF, C_2_ = 5pF, *L* = 1.3µH, R_1_ = 2.2kΩ, and R_3_ = 22kΩ. Resistor R is a potentiometer of 2kΩ, that acts as a variable resistor, whose values are tuned in the range of 1kΩ to 2kΩ for the sustained oscillation of the circuit. The circuit is run in transient analysis to obtain the chaotic waveforms. For *R* = 1650Ω, three chaotic waveforms, V_C1_, V_C2_, and I_L_, are obtained as shown in Figs. 14, 15, and 16. Figs. 14, 15, and 16 show the effectiveness of the proposed Chua chaotic oscillator for sustained oscillation.

**Fig. 17:**
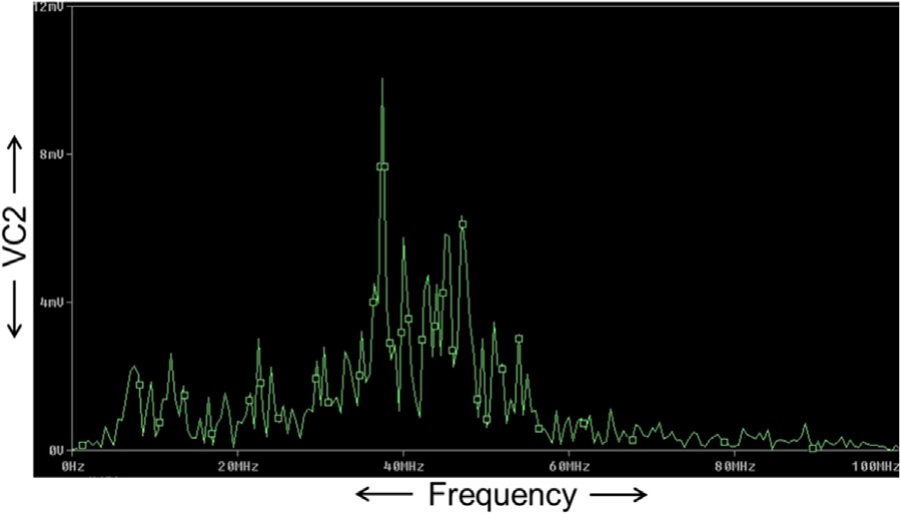
FFT of voltage chaotic waveform V_C2_.

**Fig. 18:**
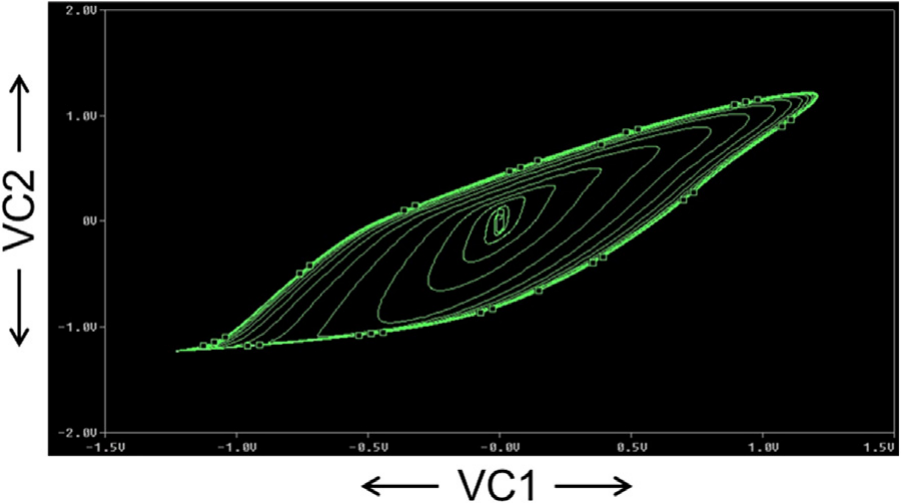
Large limit cycle obtained at *R* = 1450Ω.

**Fig. 19:**
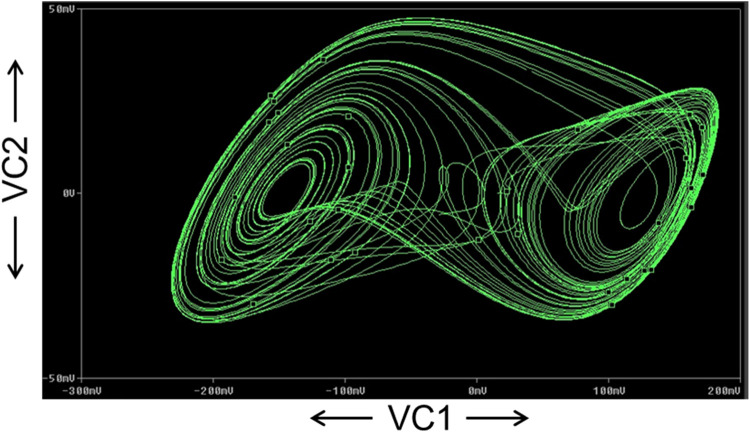
Double scroll attractor obtained at *R* = 1650Ω.

**Fig. 20:**
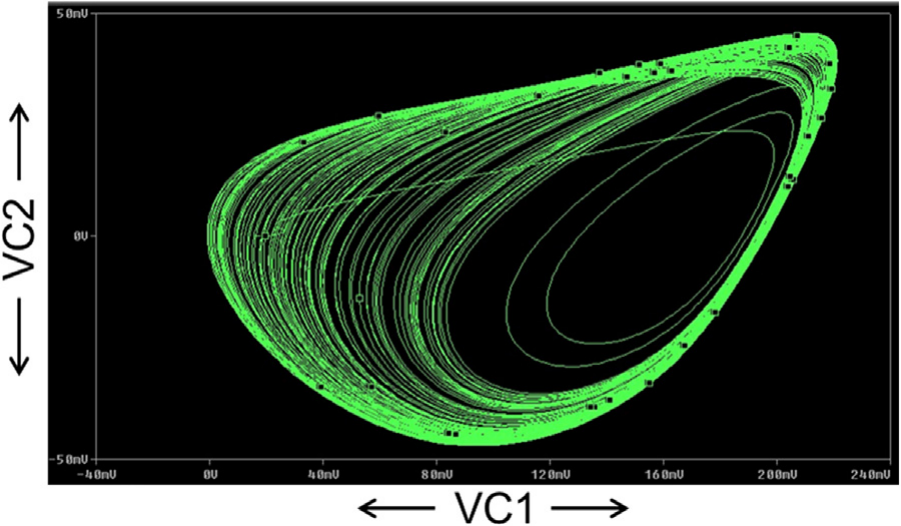
Rossler type attractor obtained at *R* = 1800Ω.

**Fig. 21:**
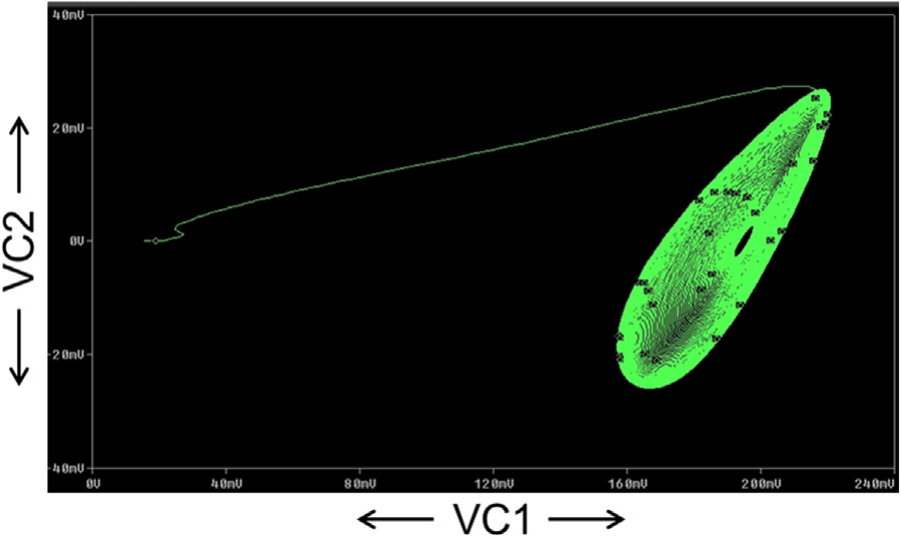
I-periodic attractor obtained at *R* = 1950Ω.

The FFT of the V_C2_ is shown in Fig. 17, which confirms that the dominant operating frequency of the proposed chaotic oscillator is 37.5MHz. Also, chaotic attractors like a large limit cycle, double-scroll attractor, Rossler-type attractor, and I-periodic attractor are obtained by varying the resistance R, as shown in Figs. 18, 19, 20, and 21, which again confirms the ability of the proposed chaotic oscillator circuit.

**Fig. 22:**
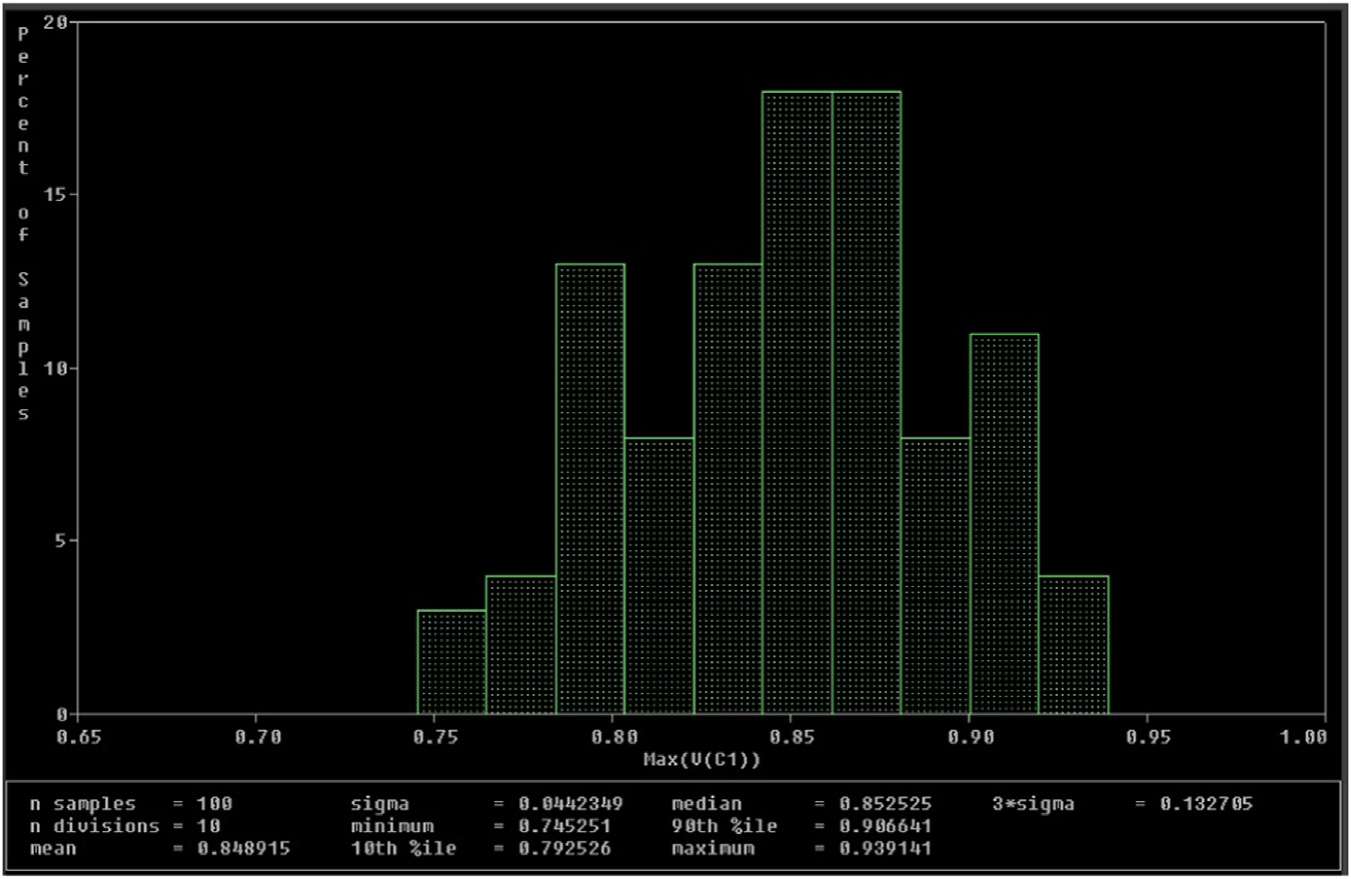
Histogram of V_C1_.

**Fig. 23:**
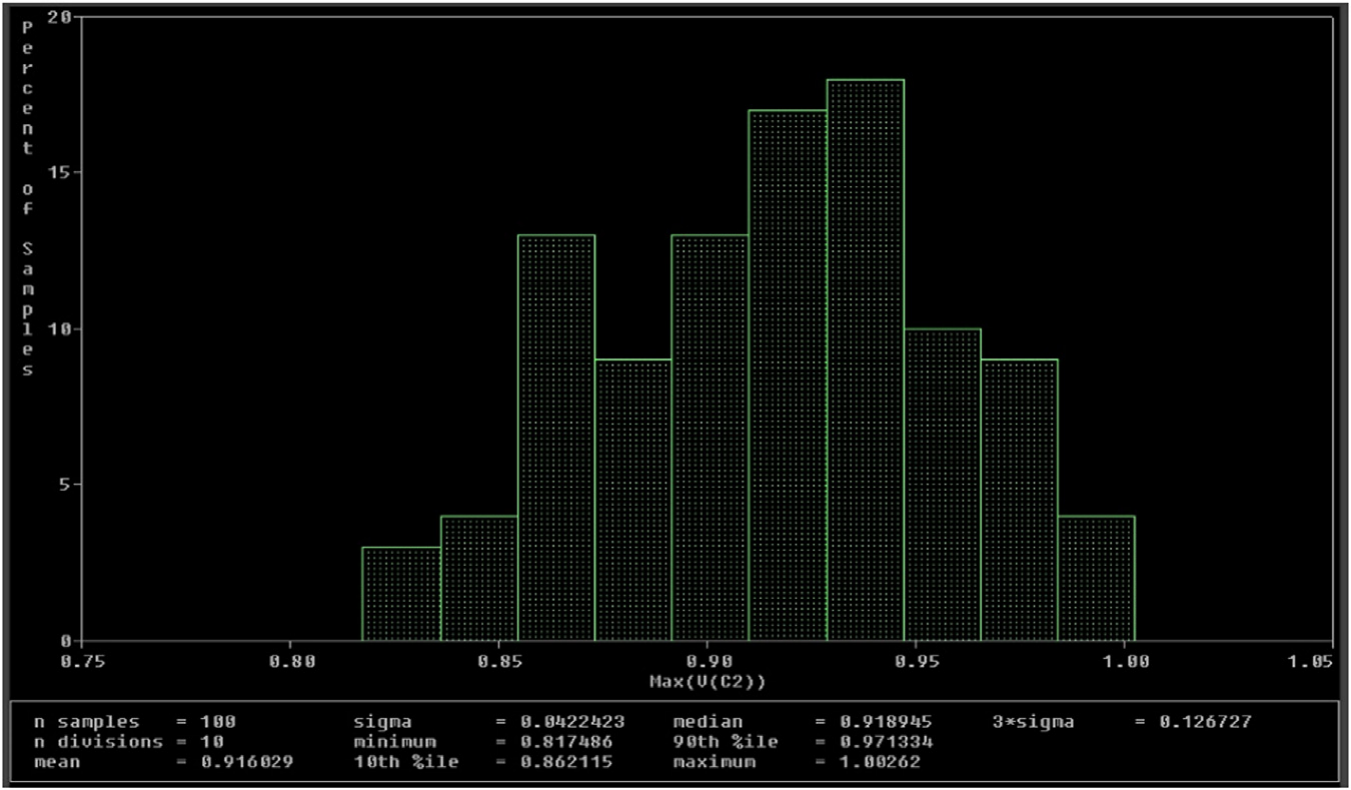
Histogram of V_C2_.

A Monte Carlo simulation has been performed to evaluate the uncertainty and robustness of the proposed Chua chaotic circuit. For this simulation, all passive components used in Fig. 13 are assigned tolerances as follows: resistors R, R1, and R3 have 5%; capacitors C1 and C2 have 7%; and the inductor L has 7%. A uniform distribution has been used for 100 runs. The VC1 and VC2 waveforms for these 100 runs are analyzed and presented in histograms, as shown in Fig. 22, Fig. 23. From Fig. 22, a deviation of 0.0442349 is observed for the mean value of 0.848915. This indicates a deviation of 5.21% for the 100 runs, which is within the acceptable range. Similarly, in Fig. 23, a deviation of 0.0422423 is observed for the mean value of 0.916029. This indicates a variation of around 4.61% across the 100 runs, also falling within the permissible bounds.

## Conclusion

A CCTA-based Chua chaotic oscillator is implemented to generate chaotic waveforms in the electronics lab. First, a straightforward Chua diode, called a non-linear resistor, is designed by combining two CCTA-based negative resistances. This Chua diode is further used in the traditional Chua circuit to obtain a high-frequency CCTA-based Chua chaotic oscillator. The proposed NR_1_, NR_2_, and Chua diode circuits are simulated using an IC-based CCTA to obtain their V-I characteristics. The proposed Chua circuit is simulated using a CMOS implementation in 180nm technology to get a high dominant operating frequency. Three chaotic waveforms, V_C1_, V_C2_, and I_L_, are obtained in the simulation. Various chaotic attractors are also obtained along with the chaotic waveforms, confirming the proposed circuits' ability to generate sustained chaotic waveforms. A Monte Carlo simulation has been performed to evaluate the uncertainty and robustness of the proposed Chua chaotic circuit and found variations falling within the acceptable range.

## Limitations

Not applicable.

## Ethics statements

This research did not involve research on human subjects or animal experiments, and no data is involved from social media platforms.

## CRediT authorship contribution statement

**Chandan Kumar Choubey:** Conceptualization, Methodology, Validation, Writing – review & editing, Writing – original draft. **Aruna Pathak:** Supervision, Visualization, Investigation. **Manoj Kumar Tiwari:** Methodology, Writing – review & editing.

## Declaration of competing interest

The authors declare that they have no known competing financial interests or personal relationships that could have appeared to influence the work reported in this paper.

## Data Availability

No data was used for the research described in the article.
